# Lignin-based nano-enabled agriculture: A mini-review

**DOI:** 10.3389/fpls.2022.976410

**Published:** 2022-10-26

**Authors:** Matteo Gigli, Guido Fellet, Laura Pilotto, Massimo Sgarzi, Luca Marchiol, Claudia Crestini

**Affiliations:** ^1^ Department of Molecular Sciences and Nanosystems, Ca’ Foscari University of Venice, Venezia-Mestre, Italy; ^2^ Department of Agricultural, Food, Environmental and Animal Sciences, University of Udine, Udine, Italy; ^3^ Department of Life Sciences, University of Trieste, Trieste, Italy

**Keywords:** lignin nanoparticles, sustainable agriculture, circular economy, nanocarriers, nano-enabled agriculture

## Abstract

Nowadays sustainable nanotechnological strategies to improve the efficiency of conventional agricultural practices are of utmost importance. As a matter of fact, the increasing use of productive factors in response to the growing food demand plays an important role in determining the environmental impact of agriculture. In this respect, low-efficiency conventional practices are becoming obsolete. On the other hand, the exploitation of nanoscaled systems for the controlled delivery of fertilizers, pesticides and herbicides shows great potential towards the development of sustainable, efficient and resilient agricultural processes, while promoting food security. In this context, lignin − especially in the form of its nanostructures − can play an important role as sustainable biomaterial for nano-enabled agricultural applications. In this review, we present and discuss the current advancements in the preparation of lignin nanoparticles for the controlled release of pesticides, herbicides, and fertilizers, as well as the latest findings in terms of plant response to their application. Special attention has been paid to the state-of-the-art literature concerning the release performance of these lignin-based nanomaterials, whose efficiency is compared with the conventional approaches. Finally, the major challenges and the future scenarios of lignin-based nano-enabled agriculture are considered.

## 1 Introduction

The food system was recognized as “the major driver of climate change, changes in land use, depletion of freshwater resources, and pollution of aquatic and terrestrial ecosystems through excessive nitrogen and phosphorus inputs” (Springmann et al., 2018). As the world population is expected to reach 9.7 billions by 2050 (UNDESA 2019), a higher demand in terms of arable land (+67%), irrigation water (+65%), and N (+51%) and P fertilizers (+54%) is required (FAO, 2009). Consequently, the environmental pressure of agriculture, already very high, will rise even more (Renner et al., 2020), due to the low efficiency of obsolete conventional agriculture practices no longer able to support food security. The current food system thus requires an agricultural revolution based on sustainable intensification and driven by system innovation (Willett et al., 2019). In this context, the field application of fertilizers, pesticides and herbicides through nanocarriers shows great promise for a sustainable, efficient and resilient agricultural system, while promoting food security (Xin et al., 2021). Similarly, the use of renewable materials to produce nanosized delivery systems, especially if deriving from waste biomass, represents another crucial step towards the green transition and the fulfillment of circular economy paradigms.

**Figure 1:**
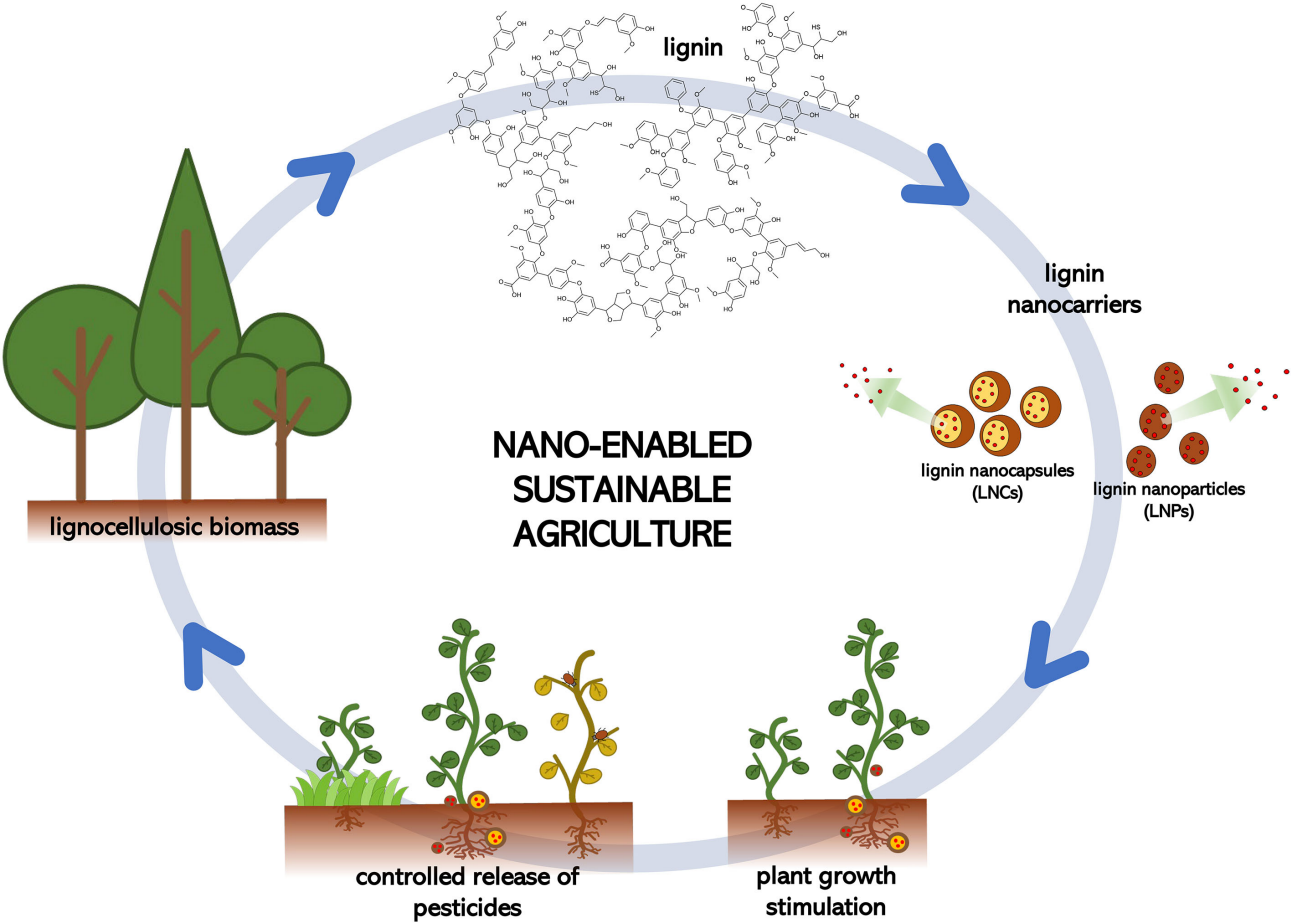
Lignin-based approach towards a nano-enabled sustainable agriculture.

In this framework, lignin represents a highly valuable, yet underutilized bioresource (Calvo-Flores et al., 2015). Over 50 Mt/y of technical lignins are obtained as byproduct of pulp and paper and modern biorefinery processes, making it largely available at low cost. Its peculiar polyphenolic structure is of high interest towards the generation of nanomaterials. Indeed, lignin a) yields nanostructures by supramolecular self-assembly, b) permits the controlled release of active substances thanks to a stimuli-responsive behavior and high chelating properties (García et al., 1996), c) possesses intrinsic antimicrobial, antioxidant and UV-shielding characteristics allowing for the protection of entrapped/encapsulated compounds, d) is of natural origin, biocompatible and biodegradable: it thus constitutes a valid alternative to synthetic polymers commonly employed in this field (Sipponen et al., 2019). Therefore, lignin-based delivery systems could significantly contribute to the development of a sustainable, nano-enabled agriculture (Ramírez et al., 1997; Sun, 2010; Lu et al., 2022) and to the reduction of microplastics (**Figure 1**).

Recent reviews (e.g.: Lima et al., 2021 and Machado et al., 2022), discuss the potentialities of lignin nanostructures in agriculture, focusing on the applications of these nanomaterials as pesticides or as carriers of pesticides. Lignin nanoparticles can be utilized as stimulators or inhibitors of the plant growth or as agents to control the release of entrapped active molecules.

In this contribution, an overview of the most relevant studies on the employment of lignin-based nanocarriers for agricultural applications is provided, to highlight their beneficial effects on crops. Finally, open questions and research needs are outlined.

## 2 Lignin-based delivery systems

**Figure 2:**
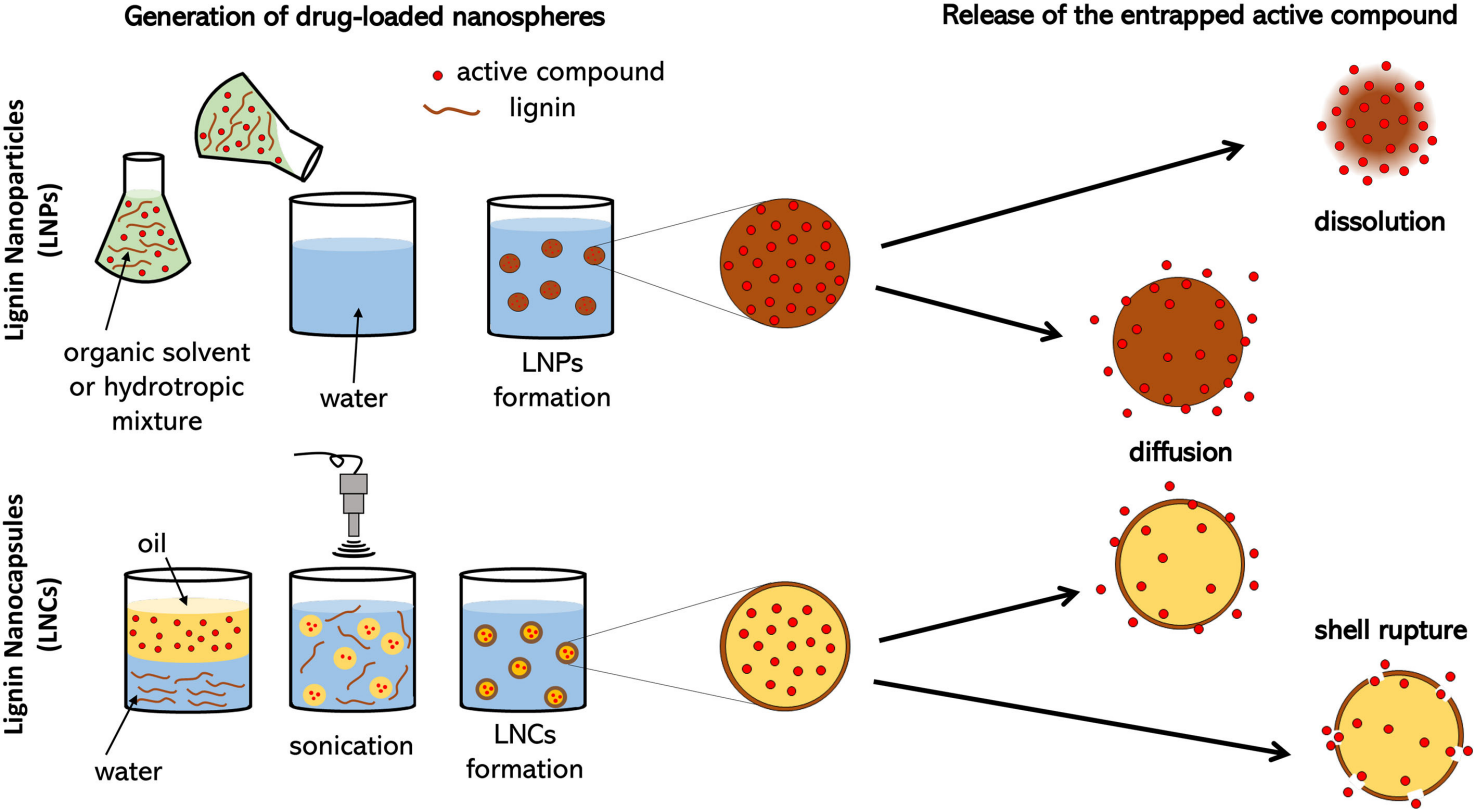
Synthetic strategies for the preparation of lignin-based nanocarriers.

Lignin-based nanocarriers can be divided into lignin nanoparticles (LNPs) and lignin nanocapsules (LNCs). Both types have a spherical shape: while LNPs are consist of full lignin matrix particles in which the active compounds can be dispersed, LNCs are hollow particles containing liquid (or solid) substances within the lignin shell. The intrinsic supramolecular self-assembly characteristics of lignin are exploited to form LNPs and LNCs *via* different synthetic strategies (**Figure 2**).

LNPs can be conveniently synthesized by sonication of appropriate lignin solutions (Gilca et al., 2015). Usually, they are generated by nanoprecipitation, i.e. dissolution in an organic solvent and subsequent dilution with water, in which lignin is insoluble (Gigli et al., 2021). Another strategy is solvent-exchange through dialysis. By varying the pre-dialysis lignin concentration, the membrane cut-off and the used solvent, the characteristics of the LNPs can be controlled to a certain extent (Lievonen et al., 2016). Alternatively, to avoid the use of organic solvents, LNPs can be prepared by dilution of a highly concentrated aqueous solution of a hydrotropic agent, an ionic organic salt promoting the dissolution of lignin. The hydrotropic agent can be easily recovered and recycled, thus enhancing the sustainability of the process (Cailotto et al., 2020).

On the other hand, LNCs are obtained from oil-water emulsions. Lignin is dissolved in the water phase or in a volatile organic solvent, while the oily phase contains the active ingredient constituting the particles’ core. Emulsification is typically achieved by ultrasounds (optionally, a surfactant can be used) and LMCs can be further stabilized by interfacial crosslinking. Recently, the formation of LMCs by simple ultrasonication was demonstrated (Gilca et al., 2015; Sgarzi et al., 2022; Zongo et al., 2019). The application of ultrasounds leads to cavitation phenomena locally generating high temperatures and pressures promoting the π-π stacking interactions of lignin aromatic groups and the establishment of intermolecular hydrogen bonds. By the modulation of the sonication intensity, it is also possible to induce chemical crosslinking and thus tune capsules shell thickness, stability and release properties.

LNPs and LNCs not only display a pH-responsive behavior (Cailotto et al., 2020; Sgarzi et al., 2022), but also the nature and concentration of salts has a significant influence on the stability of the particles (Zongo et al., 2019). More detailed information on the synthetic methodologies towards lignin-based nanostructures can be found in recent literature reviews (Sipponen et al., 2019; Chen et al., 2021).

### 2.1 Controlled release of biocides

The traditional methods for crop protection require repeated applications of large volumes of active species at high initial dosages. Moreover, the non-controlled delivery causes a time-limited biocidal protection and causes the ubiquitous presence of biocides in the environment, resulting in biocide resistance and soil/water/food chain contamination (Mattos et al., 2017; Usman et al., 2020). In this context, lignin represents a green matrix for the design of sustainable biocide delivery systems, which are an effective tool for a controlled-release and stimuli-responsive delivery. **Supplementary Table 1** summarizes the main contributions to this topic.

#### 2.1.1 Fungicides and nematicides

Kraft LNPs were used for the *in situ* growth of brochantite crystals for the controlled release of Cu^2+^ ions against *P. syringae tomato, X. campestris, X. arboricola fragari* and *B. cinerea* (Gazzurelli et al., 2020). The 10% w/w Cu^2+^-composite (ca. 300 nm) allowed to achieve a 20× reduction of used copper with respect to commercial copper hydroxide. Interestingly, the enhanced leaf adhesion of 10-30 nm stick-shaped brochantite crystals rendered them more efficient than 2-10 nm spherical ones. Aggregates of LNPs (> 600 nm) were proposed as Cu^2+^-substitutes, inhibiting the growth of *X. arboricola in vitro* and decreasing the incidence of the related disease on *Corylus avellana* with performance comparable with copper oxychloride (Schiavi et al., 2022).

Kraft methacrylated lignin was crosslinked with pyraclostrobin (>90% encapsulation efficiency, EE) to prepare nanocarriers for the treatment of Esca disease in *V. vinifera* cv. ‘Portugieser’ (Fischer et al., 2019). The degradation of lignin by the Esca fungi laccases and peroxidases triggered the controlled release. A single injection of these 200 nm-nanocarriers reduced Esca symptoms up to 5 years by utilizing only 3% of the pyraclostrobin used in conventional preventive spraying. Similar results were obtained preparing 200-300 nm spermine/spermidine crosslinked lignin nanocarriers of different fungicides (azoxystrobin, boscalid, pyraclostrobin and tebuconazole; EE 70-99%). The encapsulated species exhibited lower (2-10×) minimum inhibitory concentrations than the bulk counterparts (Machado et al., 2020).

The delivery of natural fungicides (*E. arvense, R. tinctorum, S. marianum*, and *U. dioica* extracts) was achieved *via* crosslinked lignin-chitosan nanocarriers. Differently from lignin-diamine crosslinked systems, these 185-nm spherical capsules possess large cavities able to host large bioactive compounds such as flavonoids and terpenes contained in the extracts with a 95% EE. The 90% maximal effective concentration (EC_90_) values of the encapsulated extracts against *N. parvum* were lower than the non-encapsulated ones (e.g. 90.6 μg·L^-1^ *vs* 2938 μg·L^-1^, respectively, for *S. marianum*), proving a higher antifungal activity of the former formulations (Sánchez-Hernández et al., 2022).

The delivery of the nematicide abamectin was realized *via* lignosulfonate-modified epoxy resins (EE 93.4%) (Zhang et al., 2020). The 150 nm spherical nanocapsules exhibited a slower release rate (73% after 18h) than abamectin suspensions and microemulsions (96% after 1h and 91% after 4h, respectively). Moreover, these nanocarriers improved the mobility and the distribution of abamectin in soil compared to the other formulations and exhibited a higher absorption in *Cucumis sativus* roots and in *M. incognita*.

#### 2.1.2 Insecticides

Avermectin (AVM) was loaded into 108 nm hollow spheres composed of lignin-based azo polymer (61% EE). The system exhibited 80% cumulative release after 120h in a 7:3 ethanol:water mixture (cf. AVM emulsifiable concentrate, 100% after 36h). Moreover, the UV-blocking properties of lignin increased the photostability of AVM under UV irradiation (Deng et al., 2016).

A xylanase-responsive controlled release of AVM (57.9-67% EE) was achieved by the use of lignin-xylan nanospheres (160-210 nm) (Jiang et al., 2020), which released 42% AVM in 2h. After 16h, a 55% equilibrium value was reached, 10× higher than in xylanase-free experiments.

Lignosulfonate (LS)-cetyltrimethylammonium bromide (CTAB) nanospheres were prepared for the entrapment of AVM. Cationic nanospheres (94 nm) were obtained with a 1.5:1 LS : CTAB mass ratio, while changing it to 4:1 yielded anionic nanosystems (170.8 nm). The cationic nanospheres exhibited a ca. 80% AVM release in 62h, while the anionic nanosystems released ca. 55% of AVM in the same time range (cf. AVM emulsifiable concentrate, 100% in 6h). These nanoformulations presented an enhanced (2.18-2.96×) AVM anti-photolysis activity with respect to AVM emulsifiable concentrate (Peng et al., 2020).

AVM was encapsulated in co-surfactant-free hollow nanospheres (300 nm) thanks to the self-assembly properties of alkyl chain-crosslinked lignosulfonate (Liu et al., 2017). A burst 20% release in the first 10h, ascribable to AVM adsorbed on the capsule’s surface, was observed in a 3:7 ethanol:water mixture. Only 27.6% of AVM was released after 241h due to the slow diffusion of the insecticide from the spheres’ core (cf. AVM emulsifiable concentrate, 100% after 20h, and suspension, 80% after 10h). The capsules exhibited excellent UV-shielding performance (100% AVM photolysis after 69h UV irradiation) compared to AVM emulsifiable concentrate and suspension (100% AVM photolysis after 10h and 5h UV irradiation, respectively).

To improve the adhesion and the retention of insecticides on leaves, lignin-containing cellulose nanofibers were prepared and loaded with emamectin benzoate (EB) (Zhang et al., 2022). The electrostatic interaction between the positively charged quaternized nanofibres and the negatively charged leaves increased the retention rate of the droplets of EB. The insecticidal activity was tested against *M. separata*, whose mortality increased by 35% with respect to the one of non-supported EB, and by 50% in rain-fastness tests. A 90% mortality after 1.5h UV irradiation was kept using these fibers, thanks to the UV-shielding properties of lignin (cf. 10% mortality for non-supported EB).

High EE (85.9-99.9%) were obtained for EB loaded onto lignosulfonate nanoparticles (150-250 nm) *via* electrostatic interactions. The insecticidal activity was improved thanks to the higher anti-photolysis ability (4× higher than EB emulsion), and to the lower release rate in methanolic solutions (30% and 60% lower than EB emulsion and suspension, respectively). The pH-responsiveness of this nanocarrier allowed to obtain faster EB release at acidic pH (Cui et al., 2019).

Cyhalothrin was loaded on a core-shell-shell nanostructure composed of lignosulfonate and dodecyl dimethyl benzyl ammonium chloride (DDBAC). The capsules were stabilized by the addition of iron(III) ions, which formed complexes with lignosulfonate constituting the outer shell. A concentration of 10 g·L^-1^ DDBAC yielded 216 nm nanocarriers with a 94.5% EE. Encapsulated cyhalothrin half-life was increased by 4.4 times under UV irradiation with respect to the emulsifiable concentrate. Alkaline pH and laccase could increase cyhalothrin release rate (Zhang et al., 2021).

Lignin-based pH-responsive nanogels were employed to prepare chlorpyrifos carriers. Lignin methacrylate was graft-copolymerized on acrylic acid to yield a 113 nm-core-shell spherical structure. The slowest release of chlorpyrifos (ca. 45%) was achieved at pH 7 in 7h using the nanogel prepared with a 40 mg·mL^-1^ acrylic acid formulation (Yiamsawas et al., 2021).

The translocation of methoxyphenoxide (MFZ) in *Glycine max* under hydroponic conditions was enhanced by its encapsulation in 113.8 nm LNPs grafted to poly (lactic-*co*-glycolic) acid. This 2.7% w/w MFZ core-shell nanosystem exhibited a temperature-dependent release behavior (90% MFZ release after 80h at 25°C, 100% release at 37°C after 20h). The encapsulated MFZ was efficiently translocated to the roots and, differently from non-encapsulated MFZ, its concentration increased over time. Thanks to the LNPs negative charge, their translocation efficiency resulted relatively higher than particles of similar size reported in the literature, and decreased with increasing their concentration (after 24h, 0.065 for 0.01 mg·mL^-1^ LNPs and 0.006 for 0.1 mg·mL^-1^ LNPs). Compared to non-encapsulated MFZ, LNPs were able to deliver 7-17-fold more MFZ (Mendez et al., 2022).

#### 2.1.3 Herbicides

Subabul stem lignin was utilized to produce a 74.3% EE diuron nanoformulation (Yearla and Padmasree, 2016). The 150-190 nm LNPs exhibited an initial burst release (25%), followed by a relatively slower regime. The increase of pH increased diuron release rate: the behavior at pH 7 and 9 resulted similar (67% and 62%, respectively, after 120 days) and higher than the one registered at pH 5 (53%). This nanoformulation presented excellent performance compared to commercial diuron formulation and bulk solid (100% diuron in 2 days), and induced a more pronounced leaf mortality and chlorosis on *B. rapa*.

### 2.2 Stimulation of plant growth

Other studies focused on the release of fertilizers and on the impact of lignin-based nanomaterials on the health of various plant species (Falsini et al., 2019; Yin et al., 2020; Del Buono et al., 2021; Salinas et al., 2021). The possible adverse effects and the ability to stimulate plant growth were evaluated, either using lignin nanocarriers for the delivery of bioactive compounds or assessing whether LNPs themselves could exert beneficial effects on seed germination and seedlings development (**Supplementary Table 1**).

Lignin-based magnetic nanoparticles (M/ALFeP) were used as phosphorus adsorbent in wastewater and subsequently as slow-release nano-fertilizer (Li et al., 2021). Fe_3_O_4_ nanoparticles were embedded in alkaline amino-functionalized lignin and chelated with Fe(III) ions. The so-formed hybrid nanoparticles (300-700 nm) adsorbed ${\text{HPO}}_{4}^{-}$ following a pseudo-second-order kinetic model with maximum adsorption capacity and removal rate at pH 9 (q_e_ = 12.8 mg·g^-1^ with 50 mg·mL^-1^ ${\text{H}}_{2}{\text{PO}}_{4}^{-}$ and 20 mg·mL^-1^ nanoparticles). M/ALFeP sustained the release of both Fe (67.2%) and phosphorous (69.1%) over 30 days at neutral pH at RT. The recyclability of the prepared nanoparticles was also demonstrated, the recovery and removal efficiency being respectively 83% and 62% as compared to the freshly prepared M/ALFeP.

The effect of lignin-graft-poly(lactic-*co*-glycolic) nanoparticles (ca. 100 nm), prepared by emulsion evaporation, on the germination of soybean seeds was investigated (Salinas et al., 2021). No significant variations versus the control were observed in terms of chlorophyll content, root and stem length upon exposure to LNPs (0.02, 0.2, 2.00 mg·mL^-1^), although the root biomass increased at higher doses. B, S, and Mo uptake exhibited no variations, while high concentrations of Na and Zn were found in the seedling roots at high LNPs doses. The increased Na concentration was well tolerated by soybean, thanks to its metabolic and structural adaptation mechanisms (Phang et al., 2008). Lastly, the activity of superoxide dismutase in the leaves increased after 7d for all LNPs doses, proving a minimum LNPs-induced oxidative stress.

In another study, kraft lignin-based LNCs (200 – 250 nm) were prepared by sonication and loaded with gibberellic acid (GA3), a seed germination enhancer (Falsini et al., 2019). *Eruca vesicaria* (arugula) and *Solanum lycopersicum* (tomato) seeds were treated with 0.5, 1.0, and 1.5 mg·mL^-1^ GA3 formulations, both in solution and encapsulated in LNCs, and with GA3-free LNCs. The presence of either GA3-loaded or GA3-free LNCs resulted in an increase of germinated seeds of arugula compared to the control. Furthermore, GA3-containing LNCs boosted the growth of stem and root length, and of vegetative biomass. As to the tomato, no positive impacts were detected for stem and root length of seedlings treated with LNCs, while GA3-loaded LNCs enhanced seed germination. LNCs were found in the endosperm of tomato seeds and in the cortex layer of the germinated roots reaching the xylem vessels after 72h, confirming LNCs permeation and accumulation. These effects were ascribed to the abundance of hydroxyl groups on the surface of LNCs that may promote seeds germination and growth by enhancing water availability.

Abscisic acid (ABA), a UV-sensitive plant growth regulator, was entrapped in LNPs (ca. 300 nm) to achieve its controlled release and increase its photostability (Yin et al., 2020). CTAB was used to limit the formation of aggregates. The LNPs showed a EE > 70% and a much slower release rate as compared to the control (35.5% *vs* 90% over 72h). The entrapment of ABA significantly increased its stability, as more less than 25% ABA was degraded after 60h of irradiation (cf. ca. 80% free ABA degradation in 5h). The controlled release of ABA, which is responsible for the control of stomatal aperture in case of drought stress, endowed *Arabidopsis* plants with drought-resistance ability, resulting in healthier plants as compared to those treated with free ABA.

Recently, the beneficial effects of pure LNPs (ca. 50 nm) on the germination of maize seeds was assessed (Del Buono et al., 2021). Concentrations of LNPs between 321 and 5000 mg·mL^-1^ stimulated the seed germination, while lower and higher concentrations displayed no and negative effects, respectively. 80 and 312 mg·L^-1^ dosages also enhanced the content of chlorophyll a (50%) and b (40%), of carotenoids and soluble proteins (especially 80 mg·L^-1^) with respect to the control and higher dosages. These effects were ascribed to the hormone-like action of lignin due to its phenolic structure capable of stimulating the early biochemical activities of seeds.

## 3 Conclusions and outlook

Literature production on lignin-based nanosystems for agricultural applications is so far rather limited especially to the first plant development stages, which are most likely to reveal toxicity effects. Moreover, the published works are based on lab scale experiments carried out under controlled conditions. Nevertheless, many are the involved fields of investigation, confirming both the infancy of these studies and the great potential of lignin nanocarriers as bio- and eco-compatible materials for sustainable agriculture. The use of LNCs and LNPs as vectors of fertilizers and active molecules needs further investigations, not only to define the doses and the efficacies, but also to verify their environmental sustainability.

Moreover, the specific mechanisms for the nanovectors uptake and translocation in plants are still not well understood and plant-dependent interactions with these new formulations have to be considered. Additionally, the type of administration (e.g. foliar *vs* soil application) might also affect the influence on the soil biology and the soil-plant interaction.

Altogether, greenhouse scale and ultimately field scale experiments are necessary to validate the advantage of using lignin nanocarriers *vs* traditional approaches for the entire plant cycle from sowing to harvesting. The gap between the lab experiments and the open field trials is still wide, but also very rich in research opportunities for the transition toward a more sustainable agriculture.

## Author contributions

MG and GF have contributed equally to this work and share first authorship. All authors contributed to the article and approved the submitted version.

## Funding

Ca’ Foscari University, FPI (Fondi primo insediamento) 2019, grant number H74I19001780005.

## Acknowledgments

The Ca’ Foscari University, FPI (Fondi primo insediamento) 2019 grant is gratefully acknowledged.

## Conflict of interest

The authors declare that the research was conducted in the absence of any commercial or financial relationships that could be construed as a potential conflict of interest.

## Publisher’s note

All claims expressed in this article are solely those of the authors and do not necessarily represent those of their affiliated organizations, or those of the publisher, the editors and the reviewers. Any product that may be evaluated in this article, or claim that may be made by its manufacturer, is not guaranteed or endorsed by the publisher.
