# Supplementary material for: Lignin-based nano-enabled agriculture: A mini-review
Source: Front Plant Sci. 2022 Oct 26;13:976410. doi: 10.3389/fpls.2022.976410 (PMC9667414; doi:10.3389/fpls.2022.976410)
Supplement: Supplementary file 1 [file Table_1.docx]

Supplementary Material

**Supplementary Table 1**: Summary of different types of lignin-based nanocarriers used for nano-enabled agricultural applications

| Use | Material | Species | Treatment | Experimental conditions | Results | Ref. |
| --- | --- | --- | --- | --- | --- | --- |
| Biostimulant | Lignin nanocapsules loaded with gibberellic acid  (200-300 nm) | *Eruca vesicaria* and *Solanum lycopersicum* | Seed priming with lignin nanocapsules (LNCs) loaded with three different concentrations of GA:  0.5 mg·mL^-1^ (NGA0.5),  1 mg·mL^-1^ (NGA1.0),  1.5 mg·mL^-1^ (NGA1.5) | Germination trials in Petri dishes | Increased plant growth (total length and dry weight). Enhanced percentage of treated seed germination respect the untreated control | Falsini *et al.* (2019) |
| Biostimulant | Alkali lignin nanomicroparticles loaded with abscisic acid (ABA) (300 nm) | *Arabidopsis thaliana* and *Oryza sativa* | 7.5 µL of suspension of nanocarrier, drug-loaded microspheres, and ABA on growth medium. | Germination trial in Petri dishes in climate chamber. Pot trial in growth chamber. | Lignin decreases the photodegradation of ABA. Reduction of seeds germinability after 72h compared to dimethyl sulfoxide and ABA suspensions. Enhancement of ABA effects also in drought trials. | Yin *et al.* (2020) |
| Biostimulant | Lignin nanoparticles (50 ± 20 nm) | *Zea mays* | Seed priming with five concentrations of LNPs:  80 mg·L^-1^ (T80), 312 mg·L^-1^ (T312), 1250 mg·L^-1^ (T1250), 5000 mg·L^-1^ (T5000), and 20,000 mg·L^-1^ (T20000). | Petri dishes hydroponics | No observed effect in T80. T312, T1250, and T5000 significantly stimulated maize germination. T20000 decreased the percentage of germination and reduced the radicle length. LNPs in T80, T312, T2500, and T500 increased chlorophyll A and B content. | Del Buono *et al.* (2021) |
| Use | Material | Species | Treatment | Experimental conditions | Results | Ref. |
| Biostimulant | Lignin nanoparticles (114 ± 3.4 nm) | *Glycine max* | 0.02 mg·mL^-1^, 0.20 mg·mL^-1^ and 2.00 mg·mL^-1^ in the growth medium | Hydroponics in growth chambers | No significant differences in chlorophyll content, and root and stem length compared to the control. Increased root mass at the highest concentration tested. Decreased value of TBARS after 14 days and increased in APX, CAT, and SOD content in leaves after 14 days. | Salinas  *et al.* (2021) |
| Herbicide | Lignin nano-capsules loaded with an herbicide (diuron)  (166± 68 nm) | *Brassica rapa* | The equivalent of 2.5 mg of diuron per pot | Pot experiment in growth chamber (25 ± 1 °C, photoperiod: light/dark 8/16 h. Light intensity: 40–50 μmol·m^−2^·s^−1^). | Significant inhibition of plants growth respect conventional diuron formulation and bulk diuron formulation. | Yearla and Padmasree (2016) |
| Insectide | Lignin nano-formulation loaded with an insecticide (emamectin benzoate) (150–250 nm) | *Zea mays* | The equivalent of 200, 40, 8, 1.6, and 0.32 ppm of emamectin benzoate solution sprayed on leaves | Mortality test in a pest control room (Temperature: 25 ± 2 °C; relative humidity: (75 ± 5)%; photoperiod: light/dark 16/8). | Lower mortality after 0 and 3 days from the beginning of the treatment with concentrations of 0.32, 1.6, and 8 ppm compared to conventional insecticide. After the 8^th^ day, all lignin nanoformulations concentrations demonstrated an insecticidal activity. | Cui *et al.* (2019) |
| Fungicide | Lignin nanocapsules loaded with fungicide (pyraclostrobin)  (200-700nm). | *Vitis vinifera* | < 10 mg fungicide encapsulated in 5 mL 1%wt LNPs solution | *In vitro* antimicrobial test and field experiment | Significant reduction of disease symptoms in plants. | Fischer  *et al.* (2019) |
| Fungicide | Lignin nanoparticles loaded with different concentrations of copper  (2-300 nm) | *Fragaria sp., Solanum lycopersicum* var. Optima | Different concentrations of copper loaded in nanoparticles tested. HMW@CuX% (X = 2, 6, and 10). Final concentration of 10 g·L^-1^ of Cu in the final sprayable suspensions. | Three pot trials. “1” in greenhouse conditions. “2” and “3” in climatic chambers | Higher disease control of HMW@Cu10% 3L·ha^-1^ treatment compared to copper hydroxide 3kg·ha^-1^ one. | Gazzurelli *et al.* (2020) |
| Use | Material | Species | Treatment | Experimental conditions | Results | Ref. |
| Fungicide | Lignin nanocapsules loaded with different fungicides  (200-300nm) | *Vitis vinifera* | 1, 5, 10, 25, and 50 μg·mL^−1^ of active principle. Average fungicide concentration 10-30 wt % (1-3 mg of drug per mL of the dispersion produced). Boscalid encapsulated in 5 mL 1%wt LNPs solution | *In vitro* antimicrobial test and field experiments | Inhibited growth of fungi. Decreased the symptoms of disease in four years of monitoring. | Machado  *et al.* (2020) |
| Nematicide | Three different types of abamectin nanocapsules with lignin-modified epoxy resin as nanocarrier | *Cucumis sativus* | 3.2, 1.6, 0.8, 0.4, 0.2, and  0.1 mg·L^−1^ of nanocapsules in larvicide trial. Pot experiment: 15 treatments of 37.5 mg·L^−1^ of nanocapsules per pot. Field experiment: 15 active ingredient mg per plant. | *In vitro* nematicide effects of nanopacapsules against *Meloidogyne incognita* larvae and eggs. Pot trials and field experiment. | No observed increase of larvae mortality with the nanolignin-coated-nanoparticles compared to abamectin nanocapsules without coating in larvae mortality trial and in pot and field experiments. | Zhang *et al.* (2020) |
| Insecticide | Lignin nanoparticles (113.8 ±3.5 nm) as delivery systems of methoxyfenozide (MFZ) | *Glycine max* | MFZ was incorporated into the LNPs (2.7% w/w). | Hydroponics | Effective and time dependent transfer of MFZ from hydroponic suspension to the roots, and translocation from the roots to the leaves. LNPs were able to enhance the translocation of non-systemic MFZ from the roots to the soybean aerial tissues in 24 h | Mendez  *et al.* (2022) |
| Fungicide | Lignin-chitosan nanocarriers loaded with different phytoextracts (185 nm) | *Vitis vinifera* | *In vitro*: 0, 6.25, 9.375, 12.5, 18.75, 25, 37.5, 50, 75, 100, 150 μg·mL^−1^ of nanoparticles loaded with phytoextracts. *In vivo:* 5 mL of chitosan-lignin nanocarriers loaded with *Rubia tinctorum* extract. 2 injections. One per month. | *In vitro* antimicrobial test and field application. | *R. tinctorum* resulted the best phytoextract against the pathogen tested. The minimum inhibitory concentration is 37.5 μg·mL^−1^. No phytotoxic effect of the treatment was observed. Decrease in foliar symptoms and enhancement of yield in treated plants. | Sánchez-Hernández *et al.* (2022) |
| Use | Material | Species | Treatment | Experimental conditions | Results | Ref. |
| Fungicide | Lignin nanoparticles (50-200 nm) | *Corylus avellana* | *In vitro*: 1, 0.5, 0.1 and  0.05% w/v. *In vivo*: 1% w/v solution sprayed on seedlings | Antimicrobial activity assessment *in vitro* and on seedlings (growth chamber). | *In vitro*: most performing concentration resulted in 1% w/v. *In vivo*: No phytotoxic effects were observed on seedlings. After 21 days post inoculation, no significant differences in the disease incidence compared with copper oxychloride, but no decreases in the disease severity. | Schiavi  *et al.* (2022) |
| Insecticide | Lignocellulosic nanofibers (LCNFs) and emamectin benzoate (EB) | *Zea Mays* | Equivalent of 0, 0.2, 0.5,  1 and 2 mg of EB | Insect mortality test with treated maize fragments of leaf. | Enhancement of death rate of *Mythimna separata* compared to the only EB, even at lower concentrations. | Zhang  *et al.* (2022) |
|  |  |  |  |  |  |  |
